# Supplementary material for: Rapid turnover of life-cycle-related genes in the brown algae
Source: Genome Biol. 2019 Feb 14;20:35. doi: 10.1186/s13059-019-1630-6 (PMC6374913; doi:10.1186/s13059-019-1630-6)
Supplement: Supplementary file 3 — Protein alignments, corresponding Gblocks html file and CDS sequences for the lineage conserved gametophyte-biased genes. (ZIP 2109 kb) [file 13059_2019_1630_MOESM3_ESM.zip › Con_GA_alignments/Ec-11_005060.aa_ali.fasta-gb.htm]

Ec-11\_005060.aa.fasta.trans.aa\_ali.fasta


## Gblocks 0.91b Results

Processed file: **Ec-11\_005060.aa.fasta.trans.aa\_ali.fasta**  
Number of sequences: **2**  
Alignment assumed to be: **Protein**  
New number of positions: **858** (selected positions are underlined in blue)

```
                         10        20        30        40        50        60
                 =========+=========+=========+=========+=========+=========+
Ec-11_005060     MLHALPPIVCLAPDLPPVTPTSSNSAGTLPPSFTKPFRSHHHHHRHQPHHTPPLLNTSRP
MSTRG.27797.1    ------------------------------------------------------------
                                                                             


                         70        80        90       100       110       120
                 =========+=========+=========+=========+=========+=========+
Ec-11_005060     RATLASAAVSEAAAAPQTLQAANSLATLGGGVGSLLSPQNNVLHANIFSSVRRSNNLPRK
MSTRG.27797.1    ------------------------------------------------------------
                                                                             


                        130       140       150       160       170       180
                 =========+=========+=========+=========+=========+=========+
Ec-11_005060     RPRTSSHPYGRPNNDFINEIPDLRLGSPAPAGVSPIRARKWDGNTSRDGGSLWGQSGVPP
MSTRG.27797.1    ------------------------------------------------------------
                                                                             


                        190       200       210       220       230       240
                 =========+=========+=========+=========+=========+=========+
Ec-11_005060     TGVIDPRTSAEPLETEWACPTSSVATSTSSLHDGFSDVFATPVPGGSRRAALQPHPSRFK
MSTRG.27797.1    ------------------------------------------------------------
                                                                             


                        250       260       270       280       290       300
                 =========+=========+=========+=========+=========+=========+
Ec-11_005060     VVPGRLSRSAEGLTRPRSGVRNYPMENLGNWPLNTTERWMKAIVTQDTSQERLDKLREIR
MSTRG.27797.1    ------------------------------------------------------------
                                                                             


                        310       320       330       340       350       360
                 =========+=========+=========+=========+=========+=========+
Ec-11_005060     LKRIAERERKGAAWTPVADSSSDSGDSYVSEEEPEVRGGDEEGREETEQEKEIRRKLKKA
MSTRG.27797.1    ------------------------------------------------------------
                                                                             


                        370       380       390       400       410       420
                 =========+=========+=========+=========+=========+=========+
Ec-11_005060     IRKKARRAKKREAEMKSRSNRGRWQTAGRHGGLVARAKASVPSLATRGDLVQLKGHADRV
MSTRG.27797.1    ------------------------------------------------------------
                                                                             


                        430       440       450       460       470       480
                 =========+=========+=========+=========+=========+=========+
Ec-11_005060     LIGKIARCWATVAEFFGVKPTHGGNEVHAALFRSQDLEALVAAFKREGELREKDEHRIAL
MSTRG.27797.1    ------------------------------------------------------------
                                                                             


                        490       500       510       520       530       540
                 =========+=========+=========+=========+=========+=========+
Ec-11_005060     KRQQFEESMAKRRSGPIAEVVKAYAAGLGYRPSTKKKHKRIVDGAGSNGSGGGGSGDGTD
MSTRG.27797.1    ------------------------------------------------------------
                                                                             


                        550       560       570       580       590       600
                 =========+=========+=========+=========+=========+=========+
Ec-11_005060     DGDTASVETVATSLSAASSLSSGEHPATDWWTDGGASNRWAMGPAVRKTLFLSLQAGAVA
MSTRG.27797.1    ------------------------------------------------------------
                                                                             


                        610       620       630       640       650       660
                 =========+=========+=========+=========+=========+=========+
Ec-11_005060     HLPLKQRRAAELPWVEGATHILLRRPKDALVACAKYGRDFQNLTAAQVVLALRAYAAQRI
MSTRG.27797.1    ------------------------------------------------------------
                                                                             


                        670       680       690       700       710       720
                 =========+=========+=========+=========+=========+=========+
Ec-11_005060     HAQWRGWGPRRRFAPKILALRRLVERERAIRVARRREAFGGWKAAHRSKMELMRGTRRAF
MSTRG.27797.1    ------------------------------------------------------------
                                                                             


                        730       740       750       760       770       780
                 =========+=========+=========+=========+=========+=========+
Ec-11_005060     RRWRIESERMAKTSALFRGTFWPLYVWRRWANYRISSRDKAKFLRKVYLTVILVRHFRAW
MSTRG.27797.1    ------------------------------------------------------------
                                                                             


                        790       800       810       820       830       840
                 =========+=========+=========+=========+=========+=========+
Ec-11_005060     NAVTETGQRLVGRADRASDTRLKTKADRLFRQRPGGHHSRHTTHGGSGSPSGPSARRDDA
MSTRG.27797.1    ------------------------------------------------------------
                                                                             


                        850       860       870       880       890       900
                 =========+=========+=========+=========+=========+=========+
Ec-11_005060     FDNRNHGAAAADDDDASRTSLGGMPEESATEPSDSPRNESRVGVEGDRTAKPQNSRGGGG
MSTRG.27797.1    ------------------------------------------------------------
                                                                             


                        910       920       930       940       950       960
                 =========+=========+=========+=========+=========+=========+
Ec-11_005060     GMRGRRSHHNGGGGDRGGSASAAAAAAASPPIAVLGRPNYVVFPGLRDTDFGGDVPDGGA
MSTRG.27797.1    ------------------------------------------------------------
                                                                             


                        970       980       990      1000      1010      1020
                 =========+=========+=========+=========+=========+=========+
Ec-11_005060     TFACEVLRDHFEKKEVLKARVNFLLYRRLGPQVLARLRIKANERQRSRYAAYVDARRVQK
MSTRG.27797.1    ------------------------------------------------------------
                                                                             


                       1030      1040      1050      1060      1070      1080
                 =========+=========+=========+=========+=========+=========+
Ec-11_005060     KYLKAWHFVVIQTAAVRRGITGEAMVMTLDQVRASMRKGSLEEDDVTGMESHPLAAGATA
MSTRG.27797.1    ------------------------------------------------------------
                                                                             


                       1090      1100      1110      1120      1130      1140
                 =========+=========+=========+=========+=========+=========+
Ec-11_005060     NFAGGVGATLGESTELGSGDLLCGVNQLDEDRKIRRRQVERTSIRSSRLVDRTVSLKEQS
MSTRG.27797.1    ------------------------------------------------------------
                                                                             


                       1150      1160      1170      1180      1190      1200
                 =========+=========+=========+=========+=========+=========+
Ec-11_005060     VQDAENTAAWVSARKVRERALAKFLIREGARARSADEKAQVFVAGFQQYAAENLVNVVAR
MSTRG.27797.1    ------------------------------------------------------------
                                                                             


                       1210      1220      1230      1240      1250      1260
                 =========+=========+=========+=========+=========+=========+
Ec-11_005060     IFYTVVDGRKSVTLRKTLRQWRSWYNIRMSVQLYNRQRLRNWLRICGRLRYLWRGMPLFR
MSTRG.27797.1    ------------------------------------------------------------
                                                                             


                       1270      1280      1290      1300      1310      1320
                 =========+=========+=========+=========+=========+=========+
Ec-11_005060     LLRVKWSVFHRLLELTNHRMLYGTPDLPATLRRRRELLLDYSRLLQERGFVPGRYAPEVL
MSTRG.27797.1    --------------------VRSPPPLIVCFRSN-----DKKKLVLVKGFVPGRYCPEAL
                                                        #####################


                       1330      1340      1350      1360      1370      1380
                 =========+=========+=========+=========+=========+=========+
Ec-11_005060     LPATLDPSALFQRWKQYTQARATARFLRCSLRRRHLHRVARVTFDGWRTGLGPRQVRRAA
MSTRG.27797.1    LPATLDPPALIQRWKQYTQARAAARFLRDTLRRRHRHRVARVAFDGWRTGLGPRQRREAA
                 ############################################################


                       1390      1400      1410      1420      1430      1440
                 =========+=========+=========+=========+=========+=========+
Ec-11_005060     EMRAAPTEAAAGATAAQSDAAAIGVPGRSSKRTLTEDVGEIIEEEEEEEEEEEEEDGGIA
MSTRG.27797.1    AMKAALAQGTGVAP-----ATS-VVSARPAAREAVEETLECLEEEQEEQDDDDGDELKAR
                 #############           ########################            


                       1450      1460      1470      1480      1490      1500
                 =========+=========+=========+=========+=========+=========+
Ec-11_005060     TWGAQTGDSRRSRRGKDGNLRQRKVSWVEARVEADLRVARRTVIAGWRSALTRTIGLRQA
MSTRG.27797.1    AHHPGVG--------SPWRRERRKVAFVEARADADLKVARRTVIAGWRSTLTRAIGLKNA
                                       ######################################


                       1510      1520      1530      1540      1550      1560
                 =========+=========+=========+=========+=========+=========+
Ec-11_005060     KHEHQLKEGARRIPTFKKFLAFHTWQATERVRTEARLLVDAFLHRGHLKFQDQEPPDMVG
MSTRG.27797.1    KQEFRLKKEARRQPTFKKFVAFHTWQATERVRTESRLLVDAFLNRGRLAFRDQEPPDMVG
                 ############################################################


                       1570      1580      1590      1600      1610      1620
                 =========+=========+=========+=========+=========+=========+
Ec-11_005060     RTNEKGSGSGLTFFRDKPVPGSNLVCEVRVMTRVGEGVVGIQLVMTGGKRTTELPVHGAA
MSTRG.27797.1    RSNERGSGSGLVFFRDKPVPGSYLVCEVRVMTRVGEGVVGVQLVMTGGKRTFELPVHGAA
                 ############################################################


                       1630      1640      1650      1660      1670      1680
                 =========+=========+=========+=========+=========+=========+
Ec-11_005060     VGENRSTRAHCFAVDAPVERLSRFECEHNDGLIERMRVVTSGGRWSPWFGERLTAIPDTA
MSTRG.27797.1    AGENRSTRAHSFSVDAPVERLSRFECEHNDGLIERVRVVTSGGRWSPWFGERLTGCPNPA
                 ############################################################


                       1690      1700      1710      1720      1730      1740
                 =========+=========+=========+=========+=========+=========+
Ec-11_005060     VLPDWHVHSLAATTAAAATAATKAATAASFASLDEVSSTEKAATALAAAEAQTTAANAPE
MSTRG.27797.1    ALPDWRHHSLAATTAAAAIAAKQASVAAALASLQEVSSAEKAAAAAAAADAEAAAAMAPA
                 ############################################################


                       1750      1760      1770      1780      1790      1800
                 =========+=========+=========+=========+=========+=========+
Ec-11_005060     WDVQKEYITGLVGVRTQERLVGLGVITRHVTNSHVFSYLWEAPDEDEGAMHDTNSESSLD
MSTRG.27797.1    WDIQKEYVTGLVGVRTQERLVGLGVITRHVTRSHVFSYLWEAPDEEEGSAKKKSSEDNST
                 ########################################################    


                       1810      1820      1830      1840      1850      1860
                 =========+=========+=========+=========+=========+=========+
Ec-11_005060     GRLSTSDKNGSNKSLASNSLTSIDGSATGERRSSSMNPSQARPPPPTVLGGEAPASGISN
MSTRG.27797.1    -KDSIGGQRCSGSSLASGGSADAGG-DVGRGGSSIATSSRSIPPS--P--GEAAAG----
                    ######################   ################                


                       1870      1880      1890      1900      1910      1920
                 =========+=========+=========+=========+=========+=========+
Ec-11_005060     GTEAGDGGDATGNRSHISPASSGGPTLSRVEAKQIKFAQEMRDREKLREESARLAKEREE
MSTRG.27797.1    RKGSGGIGDGSG---SVSASSSAGSTLSRAEAKQQKFAQEMRDRDREREERARLAREKEE
                                  ###########################################


                       1930      1940      1950      1960      1970      1980
                 =========+=========+=========+=========+=========+=========+
Ec-11_005060     EMERLMRGGKPNEDEEEEEGGEDPSSLSSPGMSATGKKRRKRGRAPPAAHEEFSCILRMR
MSTRG.27797.1    ELERLMRGGNTRE-EDEEDGGDDGASLDPSGLGVAGG-RGKRARAPPAPQVEFASMLRMR
                 ############# ######################  ######################


                       1990      2000      2010      2020      2030      2040
                 =========+=========+=========+=========+=========+=========+
Ec-11_005060     RTDARFALERSVALARRARTFTSNSANMFDDASGALSTLTVTVGLTNWLHSALLPQLVPL
MSTRG.27797.1    QTDARLALERSIALARSARTYSGNSANNFDDADGALSTLTVVVGLTSWLHAALLPQLVPL
                 ############################################################


                       2050      2060      2070      2080      2090      2100
                 =========+=========+=========+=========+=========+=========+
Ec-11_005060     PVLATTTTDMFDRGEKMLMTARATKARGNRMKNAAESMKIAKQKRGRRGVMSPRQRAQEI
MSTRG.27797.1    PVLATTTTDMFHRGEKMLMVARATKDSGNRVKNAAERMSTARKERGRRGVMSPTQRAKEI
                 ############################################################


                       2110      2120      2130      2140      2150      2160
                 =========+=========+=========+=========+=========+=========+
Ec-11_005060     RDREHIVEWEAKSAALLAKAHRQRVEADRLLDDARDRLPKVSQSVKTLQMYLEYLKLARR
MSTRG.27797.1    RDREHLVEWETKSLALLEKARRQREEGERLLDDARDRLPKVSQSVKTVRMYLEYLKLARR
                 ############################################################


                       2170      2180      2190      2200      2210      2220
                 =========+=========+=========+=========+=========+=========+
Ec-11_005060     QMELEREFGLGALRTKKPA---------ESA----VGGQLSESTSCGIEESLFDATIKSL
MSTRG.27797.1    QIELEREFGLGALRTRAAEGGGGGGAHDRGDGGGRDNGVLPLPSGCGIEESAFDATIKSL
                 ###############                      #######################


                       2230      2240      2250      2260      2270      2280
                 =========+=========+=========+=========+=========+=========+
Ec-11_005060     QHERHDDDAEGGATTRAGGGVGGMAGAGPSTQPSALTEEGASVGPELSGLGTIVSSVNPW
MSTRG.27797.1    LHERQDNDLR--TEGSGNGDVHEQQREGLDGSSSALAGEGGSIGAENSALGTLVSSVNPW
                 ########          ##########################################


                 
                 =
Ec-11_005060     S
MSTRG.27797.1    F
```

```
Parameters used
Minimum Number Of Sequences For A Conserved Position: 2
Minimum Number Of Sequences For A Flanking Position: 2
Maximum Number Of Contiguous Nonconserved Positions: 8
Minimum Length Of A Block: 10
Allowed Gap Positions: None
Use Similarity Matrices: Yes
```

```
Flank positions of the 10 selected block(s)
Flanks: [1300  1393]  [1405  1428]  [1463  1796]  [1804  1825]  [1829  1844]  [1878  1933]  [1935  1956]  [1959  2175]  [2198  2228]  [2239  2280]  

New number of positions in Ec-11_005060.aa.fasta.trans.aa_ali.fasta-gb:  858  (37% of the original 2281 positions)
```
